# Applicability of Retrospective and Prospective Gender Scores for Clinical and Health Data: Protocol for a Scoping Review

**DOI:** 10.2196/57669

**Published:** 2025-01-20

**Authors:** Lea Schindler, Hilke Beelich, Selina Röll, Elpiniki Katsari, Sylvia Stracke, Dagmar Waltemath

**Affiliations:** 1 Medical Informatics Laboratory University Medicine Greifswald Greifswald Germany; 2 Heart Surgery University Medicine Greifswald Greifswald Germany; 3 Internal Medicine University Medicine Greifswald Greifswald Germany; 4 Core Unit Data Integration Center University Medicine Greifswald Greifswald Germany

**Keywords:** gender score, gender medicine, medical informatics, data integration, gender health gap

## Abstract

**Background:**

Gender is known to have a strong influence on human health and disease. Despite its relevance to treatment and outcome, gender is insufficiently considered in current health research. One hindering factor is the poor representation of gender information in clinical and health (meta) data.

**Objective:**

We aim to conduct a scoping review of the literature describing gender scores. The review will provide insights into the current application of gender scores in clinical and health settings. The protocol describes how relevant literature will be identified and how gender scores will be evaluated concerning applicability and usability in scientific investigations.

**Methods:**

Our scoping review follows the PRISMA-ScR (Preferred Reporting Items for Systematic Reviews and Meta-Analyses extension for Scoping Reviews) guidelines. A title and abstract screening was conducted on PubMed, followed by a full-text screening. The inclusion and exclusion criteria were discussed by a team of 5 domain experts, and a data-charting form was developed. The charted data will be categorized, summarized, and analyzed based on the research questions during the scoping review.

**Results:**

We will report our research results according to the PRISMA-ScR guidelines. The literature retrieval was carried out on June 13, 2024, and resulted in 1202 matches. As of July 2024, the scoping review is in the data extraction phase and we expect to complete and publish the results in the first quarter of 2025.

**Conclusions:**

The scoping review lays the foundation for a retrospective gender assessment by identifying scores that can be applied to existing large-scale datasets. Moreover, it will help to formulate recommendations for standardized gender scores in future investigations.

**International Registered Report Identifier (IRRID):**

DERR1-10.2196/57669

## Introduction

The interaction of sex and gender plays an important role in the symptoms, diagnosis, therapy, outcome, life expectancy, and generally in the health and illness of men and women [1-3]. Gender includes awareness of diverse sexual orientations and identities and their relevance to daily life, including LGBTQ+ (lesbian, gay, bisexual, transgender, queer, and numerous other gender identities and sexual orientations) individuals. Neglecting or inadequately considering gender aspects in medical research and practice can have serious consequences for diagnosis, treatment, or risk prediction.

The German Ethics Council defines biological sex by genetic, hormonal, and anatomical conditions or markers, more precisely, by chromosomes, hormones, and internal and external sex organs. Gender, on the other hand, is defined by social attributes, an interaction of biological and psychosocial factors, an individual’s social biography, and is shaped by someone’s role in society. Psychological attributes determine the individual’s self-perception and sexual identity. The sexual identity may differ from someone’s physical appearance or biological sex [4]

Despite the known importance of gender in health, such as diagnostic and therapeutic responses, clinical manifestations, and disease progression, gender is currently not sufficiently represented in the health sciences, and sex and gender effects cannot yet be distinguished for most diseases [5]. The work of Vader et al [6], for instance, highlights the importance of gender and shows that gender can be seen as a mediator of sex differences. This particularly concerns chronic diseases with a higher prevalence in women (arthritis, chronic pain, and migraine). The authors even claim, that in these diseases, sex differences would not exist if there was no gender.

To improve the validity and reliability of medical research, it is necessary to encode gender information, in addition to biological sex information. We argue that a systematic gender assessment should be mandatory in future scientific investigations and in particular in clinical and health studies. Adding gender to existing data requires a retrospectively applicable score, ideally suitable for a wide range of applications. Moreover, future health research requires reliable and valid gender scores capable of predicting gender with high accuracy. The scoping review will provide an initial overview of existing gender scores and how they are currently being used in the health sciences. The objectives of this review are (1) to identify and characterize existing gender scores reported in the literature in the health sciences and (2) to systematically assess their applicability to clinical and health research (restricted to selected use cases).

## Methods

### Design

The scoping review method, as defined by Grant and Booth [7], was selected to provide an overview of the topic and to map the existing literature on the problem. It is well suited because our study deals with heterogeneous methods—covering prospective and retrospective scores, developed using strongly differing approaches—and includes a broad selection of research domains, as all subdomains of health research (eg, psychology and epidemiology) are considered. Furthermore, our review will inform future investigations on the topic [8]. In general, we followed the stepwise approach for scoping reviews described by Mak and Thomas [9] and adapted as many characteristics of the systematic review method as possible [10]. In particular, the review follows the PRISMA-ScR (Preferred Reporting Items for Systematic Reviews and Meta-Analyses extension for Scoping Reviews) guidelines [8], and the following steps were carried out: (1) identification of research questions; (2) identification of relevant studies; (3) study selection; (4) data extraction and charting; and (5) collating, summarizing, and reporting the results.

Due to limited resources, the coding of articles was conducted by a single reviewer and based on the database PubMed. It was selected as it is widely used as a standard database for biomedical literature research and is recommended as one of the most important databases for comprehensive systematic reviews [11].

### Stage 1: Identification of Research Questions

As outlined in the Introduction section, gender is currently inadequately reflected in most scientific studies. The research questions therefore arose from an initiative to standardize gender encoding in large-scale data collections maintained by the Medical Informatics Initiative Germany (MII; *Medizininformatik-Initiative*) [12] and the Study of Health in Pomerania (SHIP) [13], and to establish recommendations for standardized gender encodings in routine data provided by the Data Integration Center (*Datenintegrationszentrum*) at the University Medicine Greifswald, Germany [14].

The research questions themselves were formulated by 5 domain experts, 3 with a primary background in medicine, 1 with a background in medical informatics, and 1 with a background in medical documentation. The identified and agreed upon research questions for the scoping review are the following: (1) What gender scores exist in medicine? (2) What gender scores are applicable to the SHIP and MII data? (3) How are gender scores currently applied, or are they purely theoretical constructs?

### Stage 2: Identification of Relevant Studies

For the first assessment of the topic, a small number of relevant papers [15-17], identified through an initial manual search, were examined to identify keywords and important phrases. Furthermore, literature regarding gender awareness in the medical context [18-20] was examined to identify commonly used scientific terminology regarding gender. While this work defines a gender score as a method for assessing gender, the initial analysis showed that different terminology, such as gender measure or gender index, is used to refer to gender scores in the literature. Therefore, we included these terms in our query and added further terms as synonyms to achieve broad coverage. English language and status as a peer-reviewed article were additional inclusion criteria for our review. Furthermore, we only considered scores applied to adults and excluded scores targeting children.

**Figure 1:**
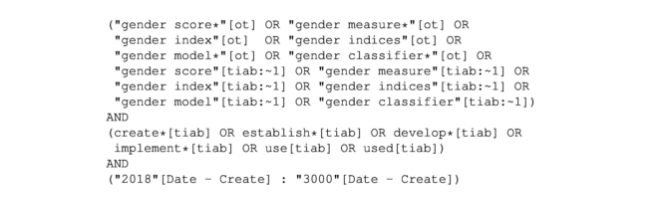
PubMed query. The query consists of three parts: (1) terminology expressed for gender score, (2) word expressing that the score was developed, (3) timeline restriction (see stage 2 for further explanation); (ot) matches keywords; (tiab) matches title and abstract; (*) wildcards match different word endings; (~1) this proximity search with distance 1 allows another word between the searched terms (different word endings are automatically included).

Based on the extracted keywords, a query was designed to capture the key elements that identify these studies. The query was optimized for recall regarding the initial literature while considering the amount of results. Specifically, the query was designed using Boolean operators, wildcards, proximity search, and a timeline. The time limit was introduced to identify more recent literature and to exclude obsolete research, such as the well-known Bem Sex-Role Inventory [21] which was shown to be outdated by Donnelly and Twenge [22]. The timeline was determined based on the distribution of articles, which showed that most research on the topic was published in the last 5 years. Throughout the entire review process, secondary literature relevant to our topic was collected. This particularly concerns older scores, which allows for the identification of earlier published literature that is still applied or cited in more recent publications. Although excluded by our initial time limit, we decided to consider them as secondary literature to evaluate if they are still reasonable to apply or obsolete. The final query was executed on June 13, 2024, and is illustrated in Figure 1.

### Stage 3: Study Selection

The obtained results were screened by title and abstract to exclude studies that do not relate to gender scores. Articles that could not be clearly excluded based on title or abstract alone were retained for further examination. The final relevance decision for the remaining articles was based on an examination of the full-text document.

### Stage 4: Data Extraction and Charting

We developed a data-charting form that was used to determine eligible data extraction points. The form was based on Gierend et al [23] and then adjusted together with the domain experts involved in this study (see stage 2). It was iteratively optimized and continuously updated throughout the process. The charting form is organized into 4 main sections corresponding to an overview and the 3 research questions. The final data-charting form is illustrated and explained in Table 1. Based on the data extraction points, 1 author critically examined the papers and extracted the relevant data. Initially, more than 30% (7/22) of the publications were randomly double-checked by a second domain expert to ensure high data extraction quality. The 2 reviewers were in full agreement on all reviewed articles. Therefore, the remaining articles are reviewed by a single reviewer throughout the remainder of the review process. Irrelevant papers are removed from the charting form.

**Table 1:** Summary of data charting.

Section		Description
**Section 1: Overview**		Summary of the basic publication information
	Article metadata	Title, Digital Object Identifier, year of publication (YYYY), keywords
	Corresponding author information	Name, research discipline, institute, Research Organization Registry (registry of open persistent identifiers for research organizations)
	Publication type	Peer-reviewed or preprint
	Study type	Gender score development, use case, or recommendation
	Continent	Continents where the study took place or focused on
	Funding source	Public, industry, none, or missing
	Citation count	Citation count as reported by PubMed and Google Scholar
	Objective	Aim or objective of the publication
	Methods	The procedures or techniques used to identify, select, process, and analyze information
	Summary results	Short description of results
	Conclusion	Short description of the conclusion
**Section 2: Research questions**		Includes the research questions and the required data
	What gender scores exist in medicine?	Necessary variables for the developed gender score Retrospective or prospective application Clinical or epidemiological study Size of cohort Target variables (feminine, masculine, binary, or continuously) Model type (statistical model or questionnaire)
	What gender scores are applicable to the SHIP^a^ and MII^b^ data?	Existence of tool support or executable model Availability of raw data or code Reproducibility Validation Limitations (eg, restricted scope) Variable coverage
	How are gender scores currently applied, or are they purely theoretical constructs?	Applicability in other research domains Practical example of usage Name and number of used records or databases Expert evaluation

^a^SHIP: Study of Health in Pomerania.

^b^MII: *Medizininformatik-Initiative* (Medical Informatics Initiative Germany).

### Stage 5: Collating, Summarizing, and Reporting the Results

This scoping review aims to summarize recently developed gender scores in health research. The extent of the review is limited to PubMed, the most relevant database for medical research outcomes. We assume that most relevant publications related to health sciences are contained in the resulting dataset. The commonly applied tools for the analytical interpretation of literature such as summary statistics, charts, and figures will be applied to discuss the collected relevant data [24]. We will follow the PRISMA-ScR guidelines [8] and discuss the general interpretation of the results compared to other evidence, the overall aim of the study, effects on practice, and future research, limitations, and recommendations.

## Results

In preparation for the scoping review, a search of the database PubMed on June 13, 2024, resulted in 1202 matches. The title-abstract screening step reduced the set to 82 and 30 potentially relevant publications, respectively. It reduced the set of considered papers more than expected. Specifically, our review identified a large number of articles not directly related to the development of gender scores. On the one hand, this is due to the positive trend that gender receives increasing attention in current research. Many articles explicitly consider gender in the form of variables or by applying existing gender scores [25-27]. These articles were matched, as we designed the query to be as inclusive as possible in order not to miss any existing scores or potential extensions of existing scores (including the terms “use” and “used” in our query). On the other hand, in the examined papers the term gender is often used to refer to binary sex categories, for example, girl or boy, female or male, or woman or man [28-32]. These papers were excluded from our review.

**Figure 2:**
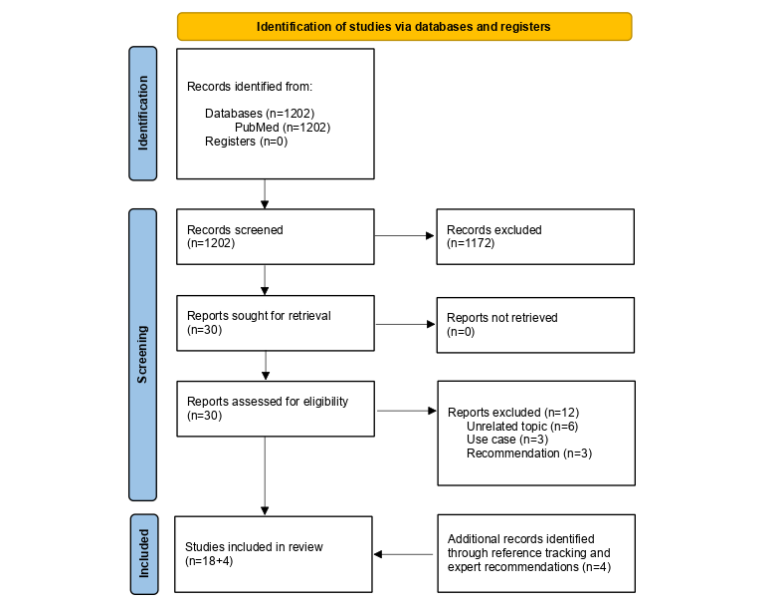
PRISMA-ScR flow diagram. The diagram illustrates the applied steps for the selection of relevant publications. PRISMA-ScR: Preferred Reporting Items for Systematic Reviews and Meta-Analyses extension for Scoping Reviews.

Full-text screening resulted in a final set of 18 papers identified in the scoping review. An additional 4 articles were identified as secondary literature, which were not covered in the timeline of the query but are still relevant in the current literature, as described earlier. A total of 22 studies were included in the review. The described process is further illustrated by the PRISMA-ScR flow diagram [8] in Figure 2. The results will be included into data charting.

We finalized the data extraction process in the third quarter of 2024 and the review will subsequently be concluded by summarizing and analyzing the results in the first quarter of 2025. The scoping review will provide an overview of recently developed gender scores in the literature. The research questions will allow us to select suited gender scores based on quality criteria and underlying research fields. The charting process will further provide information about their quality and retrospective applicability. This will serve as a basis for selecting eligible gender scores for retrospective application to existing data. Furthermore, the identified prospective scores will allow us to formulate recommendations for the inclusion of gender-dependent variables in future investigations, particularly, to incorporate standardized gender scores in investigations performed in the scope of the MII. The review will also reveal potential gaps and limitations of current gender scores, for example, regarding restrictions in underlying cohorts or research fields.

## Discussion

Our review investigates the current development and usage of gender scores in health research and aims to assess the applicability of these scores. On the one hand, the insights can help to model gender information in existing data. On the other hand, we anticipate that our data charting will help to identify gender scores that are sufficiently generic and efficient to be implemented on a large scale in future research. Furthermore, our review will provide the basis for future works on the design and development of standardized prospective approaches to gender assessments.

Our review is also a valuable addition to existing scoping reviews that address gender topics. For instance, in 2020 Horstmann et al [33] focused on recently used instruments for the operationalization of sex and gender in health research. Our review identifies gender scores that were recently developed but have not necessarily been implemented yet. We aimed to identify the latest innovations in a very active research field, so we deliberately limited our search to the last 5 years. Indeed, 13 of the 18 articles in our primary result set were published after 2020. Miani et al [34] published an overview of epidemiological aspects of gender scores. Our work, in contrast, includes gender scores from all areas of health research, such as psychology, cardiology, or neurology. Similarly to Horstmann et al [33] and Miani et al [34], we included publications until 2021.

Limitations of this work are that a single reviewer screened titles and abstracts and that our search was limited to PubMed, as explained in the Design section. However, we believe that a major strength of our review protocol is that it addresses all methods of gender assessment and that our data charting covers a wide range of information.

Appropriate consideration of gender aspects in clinical and health studies remains a fundamental issue, and we believe that further work is required to establish the standardized use of gender scores in medical data recording. While our study will provide an overview of existing gender scores, a general, broader awareness of the topic remains of high importance, and solutions need to be found to systematically apply gender scores to clinical and health studies in the future. Therefore, further effort is required to make this topic more present in the scientific community.

## Abbreviations

MII: Medizininformatik-Initiative (Medical Informatics Initiative Germany)
PRISMA-ScR: Preferred Reporting Items for Systematic Reviews and Meta-Analyses extension for Scoping Reviews
SHIP: Study of Health in Pomerania

## Data Availability

We plan to release the generated data persistently on Zenodo.
